# A Novel Heterozygous Variant in *AICDA* Impairs Ig Class Switching and Somatic Hypermutation in Human B Cells and is Associated with Autosomal Dominant HIGM2 Syndrome

**DOI:** 10.1007/s10875-024-01665-1

**Published:** 2024-02-16

**Authors:** Erika Della Mina, Katherine J. L. Jackson, Alexander J. I. Crawford, Megan L. Faulks, Karrnan Pathmanandavel, Nicolino Acquarola, Michael O’Sullivan, Tessa Kerre, Leslie Naesens, Karlien Claes, Christopher C. Goodnow, Filomeen Haerynck, Sven Kracker, Isabelle Meyts, Lloyd J. D’Orsogna, Cindy S. Ma, Stuart G. Tangye

**Affiliations:** 1 Garvan Institute of Medical Research, 384 Victoria St, Darlinghurst, NSW 2010 Australia; 2 School of Clinical Medicine, Faculty of Medicine and Health, UNSW Sydney, Sydney, Australia; 3 Department of Clinical Immunology and PathWest, Fiona Stanley Hospital, Murdoch, WA Australia; 4 Department of Immunology, Perth Children’s Hospital, Perth, WA Australia; 5 Department of Hematology, Department of Internal Medicine and Pediatrics, Ghent University, Ghent, Belgium; 6 Center for Primary Immunodeficiency Ghent (CPIG), Jeffrey Modell Diagnosis and Research Center, ERN Rita Network Center, Ghent University Hospital, Ghent, Belgium; 7 Primary Immunodeficiency Research Lab, Department of Internal Medicine and Pediatrics, Ghent University, Ghent, Belgium; 8 Laboratory of Human Lymphohematopoiesis, INSERM UMR 1163, Imagine Institute, 75015 Paris, France; 9 Université Paris Cité, 75015 Paris, France; 10 Inborn Errors of Immunity, Department of Microbiology, Immunology and Transplantation, KU Leuven, Louvain, Belgium; 11 Pediatric Immunodeficiency, Department of Pediatrics, University Hospitals Leuven, Louvain, Belgium; 12 School of Medicine, University of Western Australia, Nedlands, WA Australia

**Keywords:** HIGM2, AICDA, Ig class switching, Somatic hypermutation, Human B cells

## Abstract

B cells and their secreted antibodies are fundamental for host-defense against pathogens. The generation of high-affinity class switched antibodies results from both somatic hypermutation (SHM) of the immunoglobulin (Ig) variable region genes of the B-cell receptor and class switch recombination (CSR) which alters the Ig heavy chain constant region. Both of these processes are initiated by the enzyme activation-induced cytidine deaminase (AID), encoded by *AICDA*. Deleterious variants in *AICDA* are causal of hyper-IgM syndrome type 2 (HIGM2), a B-cell intrinsic primary immunodeficiency characterised by recurrent infections and low serum IgG and IgA levels. Biallelic variants affecting exons 2, 3 or 4 of *AICDA* have been identified that impair both CSR and SHM in patients with autosomal recessive HIGM2. Interestingly, B cells from patients with autosomal dominant HIGM2, caused by heterozygous variants (V186X, R190X) located in *AICDA* exon 5 encoding the nuclear export signal (NES) domain, show abolished CSR but variable SHM. We herein report the immunological and functional phenotype of two related patients presenting with common variable immunodeficiency who were found to have a novel heterozygous variant in *AICDA* (L189X)*.* This variant led to a truncated AID protein lacking the last 10 amino acids of the NES at the C-terminal domain. Interestingly, patients’ B cells carrying the L189X variant exhibited not only greatly impaired CSR but also SHM in vivo, as well as CSR and production of IgG and IgA in vitro. Our findings demonstrate that the NES domain of AID can be essential for SHM, as well as for CSR, thereby refining the correlation between *AICDA* genotype and SHM phenotype as well as broadening our understanding of the pathophysiology of HIGM disorders.

**Supplementary Information:**

The online version contains supplementary material available at 10.1007/s10875-024-01665-1.

## Introduction

The primary function of B cells is to recognize a diverse range of foreign antigens (Ag) expressed by pathogens or contained in vaccines, and then undergo differentiation into memory B cells and plasma cells, which contribute to long-term protection of the host against infectious diseases. The developmental pathway of a B cell from a pluripotent stem cell in the bone marrow to a differentiated effector cell in peripheral tissues is highly regulated, involving signal inputs from various cell types and immune microenvironment [1, 2]. Upon emigrating from the bone marrow, transitional B cells mature into naïve B cells expressing a unique B-cell receptor (BCR) complex comprising IgM and IgD. The presence of a functional BCR allows naïve B cells to become activated when specific Ags bind the BCR in B-cell follicles in secondary lymphoid tissues [1, 2]. After receiving help from Ag-specific CD4^+^ T cells, activated B cells undergo proliferation and Ig class switch recombination (CSR). This rearrangement process within the Ig heavy chain constant region allows B cells to switch the isotype of the clonotypic antibody from IgM/D to IgG subclasses (IgG_1_, IgG_2_, IgG_3_, IgG_4_), IgA subclasses (IgA_1_, IgA_2_), or IgE, leading to altered effector functions and tissue distribution of the secreted Ig [1, 2]. Some activated B cells will also form germinal centers (GC) in B-cell follicles, where somatic hypermutation (SHM) of the Ig variable (V) region occurs, altering the affinity of the BCR for specific Ag. This enables selection and proliferation of B cells with the highest affinity for their cognate Ag, leading to the differentiation of GC B cells into long-lived memory B cells or plasma cells and the ongoing improvement of the humoral immune response by affinity maturation [3–7].

Inborn errors of immunity (IEI) resulting from pathogenic variants in single genes that disrupt human B-cell development, selection, differentiation, and function can lead to immune dysregulatory conditions such as immunodeficiencies, autoimmunity, malignancy, and allergy [8, 9]. The study of IEI has significantly advanced our understanding of the physiological processes governing human B cell biology and humoral immunity [9, 10]. Hyper IgM syndrome (HIGM) is a rare IEI characterized by impaired CSR and SHM, resulting in absent or abnormally low levels of serum IgG, IgA, IgE, and normal or elevated levels of serum IgM. HIGM is a heterogeneous condition caused by intrinsic B cell or T cell defects that compromise the delivery or function of helper signals from CD4^+^ T cells to B cells during T-dependent B-cell activation [11–13]. Deleterious hemizygous mutations in *CD40LG*, encoding CD40 ligand (CD40L), were the first genetic aetiology of HIGM, and represents the most prevalent cause of this disease [14, 15]. CD40L is expressed on activated CD4^+^ T cells and interacts with CD40 on B cells, promoting activation, adhesion, proliferation, migration, and – in the presence of appropriate cytokines—CSR and SHM. Consequently, individuals with CD40L-deficiency are unable to form GCs, thereby preventing the generation of long-lived humoral immunity and serological memory.

Disease-causing variants in genes that intrinsically regulate CSR, SHM, and DNA repair in B cells have also been identified to cause HIGM syndrome [13]. One of these genes is *AICDA* which encodes activation-induced cytidine deaminase (AID), a 198 amino acid protein of approximately 24 kDa that is critically required for both SHM and CSR [12]. Although AID is involved in both CSR and SHM, these two processes are independent and require distinct cofactors [16, 17]. AID contains an N-terminal nuclear localization signal (NLS) and a C-terminal nuclear export signal (NES) [18]. The C-terminal domain of AID is essential to form stable dimers and associate with cofactors including RNA-binding proteins (hnRNP L, SERBP1, hnRNP U) [19, 20]. An APOBEC (apolipoprotein B mRNA-editing enzyme catalytic polypeptide)-like domain has also been described, but its exact function remains uncertain. AID contains an active site that edits DNA by deaminating deoxycytidine (dC) in single-stranded DNA to deoxyuridine (dU) resulting in U:G mismatch lesions [21]. Such mismatches are recognised and processed by DNA repair mechanisms, resulting in alterations of the DNA sequence that underlie SHM and CSR [11]. In B cells, the deamination activity of AID is concentrated at the IgV region, with the highest activity at the WRCY/RGYW (W = A/T, R = A/G, Y = C/T) hot spot motif [22], and switch (S) regions of the Ig loci to initiate SHM or CSR, respectively.

Since the first report of bi-allelic *AICDA* variants in 12 unrelated families as causal for an autosomal recessive form of HIGM syndrome (HIGM-2; AR-AID) [23], various missense and nonsense variants in exons 2, 3, or 4 of *AICDA* have been reported [23–26]. All AR *AICDA* point mutations abolished both CSR and SHM, demonstrating that the DNA-editing function of AID is essential for these fundamental processes of B-cell differentiation that generate high affinity class-switched antibodies and memory B cells. In contrast, biallelic deletion of exon 4 was recently demonstrated to encode a truncated hypomorphic AID protein, evidenced by retention of residual (fourfold reduced) SHM activity in an affected patient, but abolished CSR [25]. Unlike CD40L-deficiency, individuals with AR-AID deficiency develop lymphadenopathy, including enlarged GCs in lymphoid tissues. Interestingly, approximately 25% of individuals with AR AID deficiency also develop autoimmune phenomena, highlighting a crucial role for AID in maintaining peripheral B cell tolerance [27, 28]. Although heterozygous relatives of individuals with AR *AICDA* variants are healthy, two *AICDA* variants have been found to be pathogenic in a heterozygous state [12, 29, 30]. Both of these heterozygous variants – V186X, R190X—are located in the NES domain of *AICDA*, with the R190X variant having been identified in more than six families with autosomal dominant AID (AD-AID) deficiency [12, 16, 29–32]. These heterozygous variants truncate the C-terminal 9–13 amino acids of AID, disrupt AID intracellular localisation, and are proposed to exert a dominant negative effect on the function of the WT AID [12, 16, 29]. While these AD HIGM2 patients showed a complete impairment of CSR, the impact of pathogenic heterozygous *AICDA* variants on SHM is variable, with previous studies finding SHM to be unaffected [12, 16, 33] or significantly reduced but detectable [27, 30] in memory B cells from these individuals. This suggested that AID mediates the molecular events underpinning CSR and SHM via distinct mechanisms beyond its cytidine deaminase role. Notably, in contrast to AR-AID deficiency, none of the patients with AD AID HIGM2 have detectable levels of serum autoreactive IgM, nor do they develop autoimmune disease, and lymphadenopathy is infrequently observed [27, 28, 32]. Interestingly, most patients with AD AID deficiency present with a milder clinical phenotype than those with AR AID defects [31]. Furthermore, clinical features of individuals with the R190X variant—even within the same family—can differ dramatically, from early onset, to only ~ 40% requiring Ig replacement therapy, to asymptomatic [31].

Herein, we describe two related patients presenting with HIGM2 disease due to a novel heterozygous *AICDA* variant (c.566_568delinsAA; L189X) that leads to expression of truncated AID protein and impairs both CSR and SHM, thus further refining the correlation between AICDA genotype and SHM phenotype.

## Methods

### Ethics Statement

Buffy coats were purchased from the Australian Red Cross Blood Service. Peripheral blood was collected from AID deficient patients and their relatives. This study was approved by the Sydney Local Health District RPAH Zone Human Research Ethics Committee and Research Governance Office, Royal Prince Alfred Hospital, Camperdown, NSW, Australia (Protocol X16-0210/LNR/16/RPAH/257); and the West Australian South Metropolitan Health Service (SMHS) Human Research Ethics Committee (RGS 0665). Written informed consent was obtained from participants or their guardians. Experiments using samples from human subjects were conducted in accordance with local regulations and with the approval of the IRBs of corresponding institutions.

### Primary Cells

Peripheral blood mononuclear cells (PBMCs) were isolated by Ficoll-Hypaque centrifugation (Merck) from cytopheresis obtained from patients or healthy donors. Cells were either used fresh or cryopreserved and stored at liquid nitrogen until use.

### Overexpression of *AICDA* Variants

The HEK293T cell line was purchased from the American Type Culture Collection (ATCC) and were cultured in DMEM supplemented with 10% FBS (Gibco). HEK293T cells were grown at 37 °C, under an atmosphere containing 5% CO_2_. Empty vector (EV) and plasmids containing DDK-tagged WT *AICDA *cDNA (NM_020661.2) were obtained from Genscript. Constructs carrying single-nucleotide mutant alleles were generated from these plasmids by mutagenesis with appropriate primers, with the Q5 Site-Directed Mutagenesis kit (#E0552S, New England Biolabs), according to manufacturer’s protocol. Plasmids were amplified in competent E. coli cells (One Shot TOP10 Chemically Competent, #C404003, Thermo Fisher). HEK293T cells were plated in 6-well plates at a density of 500,000 cells per well and incubated overnight. The next day, cells transiently transfected with the various constructs in the presence of Opti-MEM (Thermo Fisher Scientific) following instructions of and Lipofectamine 3000 transfection reagent (#L3000-015, Thermo Fisher) according to the manufacturers’ instructions.

### Immunoblotting and Subcellular Fractionation

Cells were washed with cold PBS and were lysed in a buffer containing 50 mM Tris–HCl pH 7.4, 150 mM NaCl, 0.5% Triton X-100, and 2 mM EDTA supplemented with protease inhibitors (Complete Mini Protease Inhibitor Cocktail, #4,693,124,001, Roche) and phosphatase inhibitor cocktail (PhoStop, # #4,906,837,001, Roche). Lysates were incubated for 30 min at 4˚C and mixed by vortex every 10 min. The cells were centrifuged for 20 min at 16000 *g* at 4˚C, and the supernatant was collected for immunoblotting. A two-step extraction was performed to separate the cytoplasmic and nuclear content of the cells; cells were first lysed with a membrane lysis buffer (10 mM Hepes pH 7.9, 10 mM KCl, 0.1 mM EDTA, 0.1 mM EGTA, 0.05% NP40, 25 mM NaF supplemented with 1 mM PMSF, 1 mM DTT, 10 µg/ml leupeptin, 10 µg/ml aprotinin) and incubated for 30 min on ice. The lysate was centrifuged at 10,000 × *g*. The supernatant, corresponding to the cytoplasm-enriched fraction, was collected and the nuclear pellet was lysed with nuclear lysis buffer (20 mM Hepes pH 7.9, 0.4 M NaCl, 1,mM EDTA, 1, mM EGTA, 25% glycerol supplemented with 1 mM PMSF, 1 mM DTT, 10 µg/ml leupeptin, 10 µg/ml aprotinin).

Protein yield was determined with the Bradford protein assay (Bio-Rad), and equal amounts of total protein were separated by SDS-PAGE (10% polyacrylamide gel). Proteins were transferred onto a polyvinylidene difluoride (PVDF) membrane using a wet transfer system (Bio-Rad). The membrane was blocked by incubation with Intercept (PBS) Protein-Free Blocking Buffer (#927–90,001, Licor) for one hour at room temperature. Membranes were probed with antibodies directed against DDK-tag (unconjugated, clone D6W5B, #14,793, Cell Signaling), GAPDH (unconjugated, clone 6C5, #sc-32233, Santa Cruz), ACTIN (unconjugated, clone C4, #sc-32233, Santa Cruz), HISTON H3 (unconjugated, clone D1H2, #4499, Cell Signaling),. Primary antibodies were detected by incubation with goat anti-rabbit IRDye 680RD (#926–68,071, Licor) and donkey anti-mouse IRDye 800CW (#926–32,212, Licor). Binding was detected with Odyssey CLx Imager (Licor). The Chameleon Duo Prestained Protein Ladder (#928–60,000, Licor) was used to provide molecular weight marker. Images were analysed with Image studio software (Licor).

### AID Enzymatic Assay

Cytidine deaminase activity was measured using Cytidine Deaminase Assay Kit (ab239723; Abcam) following manufacturer’s instructions. Briefly, this assay uses cytidine deaminase to convert cytidine to uridine and NH_3_, as intermediates. The intermediate products then react with a proprietary reaction mix to generate a stable fluorophore that can be detected fluorometrically (Ex/Em = 410/470 nm). Total cell lysate from HEK293T cells transiently transfected with various expression vectors was performed following kit instructions. 40ug of whole cell lysate was plated in each well for cytidine deaminase activity quantification.

### Deep Immunophenotyping

Cryopreserved PBMCs and their subpopulations were analysed with a 28-color flow cytometry panel, as previously described [34]. The following mAbs were used: anti-CD20 BUV805, anti-CD10 APC, anti-Vαβ TCR BUV737, anti-CD4 APCCy7, anti-CD25 PECy7, anti-CD27 PECy7, anti-CD27 PE, anti-CD45RA PerCpCy5, anti- CXCR5 BUV615, anti-IgG APC, anti IgG BB660, anti-IgD BV480, anti-IgG BV605, anti-IgA1/A2 PECy5, anti-CD8 BUV496, anti-CD21 BUV563, anti-PD1 BV605, anti-IgM PerCPCy5.5, anti-IgM APC R700, anti-CD3 BV421, anti-IL-2 BV711, anti-IL-9 PerCPCy5.5, anti-IL-13 BV421, anti-IL-17F BV786, anti-IFN-γ BV605, anti-TNF-α BUV395, anti-CD19 BV711, anti-CD34 FITC, anti-CCR6 PE, anti-CD45RA BUV395, anti-CXCR5 BV615 (all from Becton Dickinson); anti-CD20 Pacific Blue, anti-CCR7 PECy7, anti-CD127 BV650, anti-IL-17A APCCy7, anti-CD20 BUV805, anti-CXCR3 BV421, anti-CD3 BV570 (BioLegend); (OptiBuild); anti-CCR7 FITC (R&D Systems); anti-IL-4 PECy7, anti-IL-21 e660, anti-IL-22 PE (Thermo Fisher Scientific).

### Isolation and Functional Characterization of Human T cells

Naive and memory CD4 + T cells were isolated by excluding Tregs (CD25^hi^CD127^lo^) and then sorting CD45RA^+^CCR7^+^ cells and CD45RA^−^CCR7^+/−^ cells, respectively, ensuring > 98% purity of the recovered populations. Sorted naive and memory CD4^+^ T cells were cultured in 96-well round-bottom plates (40 × 10^3^ cells/ well) with TAE beads (anti-CD2/CD3/CD28 mAb; Miltenyi Biotech). After 5 days, intracellular cytokine expression was determined following re-stimulation of cells with PMA and ionomycin for 6 hrs, with addition of Brefeldin A (10 μg/ml) after 2 h.

### In Vitro Stimulation and Analysis of Human B Cells

PBMCs were labelled with mAbs against CD20, CD10, CD27, and IgG, and sorted for transitional, naive, or memory B cells using the FACSAria III (Becton Dickinson), ensuring > 98% purity of the recovered populations. Transitional, naive, or memory B cells were cultured in 96-well round-bottom plates (30–40 × 10^3^ cells per well for CFSE analysis or 5 × 10^3^ cells per well to determine Ig secretion). B cells were stimulated with 200 ng/ml CD40L cross-linked to 50 ng/ml HA Peptide mAb (R&D Systems) alone or together with 50 ng/ml IL-21 (PeproTech) and/or IL-4 (100 U/mL; provided by R. de Waal Malefyt). B cell viability was determined using the Zombie Aqua Viability dye (BioLegend) and proliferation was measured by CFSE (eBioscience) as described previously [35, 36]. Secretion of IgM, IgG, and IgA by in vitro cultured human transitional, naive, and memory B cells was determined using Ig heavy-chain specific ELISAs, as described previously [37].

### Detection of Sars-CoV-2‐Specific B Cells

Detection of SARS-CoV-2 specific B-cells was performed as previously described [38]. Briefly, biotinylated full-length SARS-CoV-2 Spike protein was purchased from Acro Biosystems. Spike-specific B cells were identified using two fluorochromes for each protein. Thus, the biotinylated spike was incubated with streptavidin (SA)-BUV395 (BD Bioscience) or SA-PE (BD Bioscience) at a 20:1 ratio for 1 h at 4˚C. SA-FITC was used as a decoy probe to minimize background. 0.5–2 × 10^6^ previously frozen PBMCs samples were prepared and stained with 200 ng Spike and 20 ng of decoy probe in Brilliant Buffer for 1 h at 4 °C. For each experiment, PBMCs from healthy donors collected prior to 2020 (ie pre-pandemic) or from a known SARS-CoV2-exposed healthy donor were included as negative and positive controls, respectively, to ensure consistent sensitivity and specificity of the assay.

### *IGH* Repertoire Sequencing and Analysis

 Ig heavy chain (IGH) repertoires were generated from mRNA transcripts as previously described [39]. Briefly, total mRNA was extracted from FACs sorted populations of transitional, naïve and memory B cells and reverse transcribed to cDNA with oligo-dT primers (Bio-Rad). IgM (transitional, naïve and memory), IgA (memory) and IgG (memory) transcripts were amplified by PCR using forward primers binding to the leader sequences of the variable (*IGHV*) genes and a reverse primer binding to the *CH1* exon of either IgM, IgG or IgA. Samples were indexed with the Nextera Index kit and pooled into a single library which was sequenced on an Illumina MiSeq in 2 × 300 pair end format.

Demultiplexed FASTQ files were obtained from Illumina Basespace and paired using the FLASH tool [40]. Paired reads were quality filtered (minimum q20) using the FilterSeq function from the pRESTO toolkit [41] and output to FASTA format. The forward and reverse primers were trimmed with pRESTO's MaskPrimer function retaining only reads with exact primer matches (–maxerror 0). IGH constant region exons were tagged, but not trimmed, with the MaskPrimer function. Finally, pRESTO's CollapseSeq function was used to dereplicate each dataset to unique sequences retaining the read count for each unique sequence. Unique sequences were aligned against the human IMGT Reference Directory sets for *IGHV, IGHD* and *IGHJ* (obtained January 2020) [42] with standalone IgBLAST (version 1.14) [43] and output as AIRR-C tab-delimited files. IgBLAST output was filtered to require sequences have an IGHV, IGHJ and a CDR3 called, were productive (no stop codons and in-frame), were of the same isotype as the reverse primer, and had an IGHV length at least 250 nucleotides. Unique sequences that were supported by fewer than 3 reads were removed from analysis. For each individual, sequences were grouped into clonal lineages by first sub-setting the CDR3 nucleotide sequences by shared CDR3 length, IGHV (no allele) and IGHJ (no allele) and then clustering with cd-hit-est [44] at a 90% identity threshold. Amplifications from memory B cells were split into IgM^+^ memory (IgM) and switched memory (IgG/IgA) based on the isotype usage. For SHM, lineages were summarised as median SHM for all reads within the clone group for each cell type/isotype. To analyse mutation targeting, the unique sequence with the highest read count was selected from each clone and mutations relative to germline IGHVs were extracted using a perl script. Finally, repertoire features and SHM were analysed and visualised using R (version 4.3.0) [45] in RStudio (version 2023.6.2.561) [46] with the tidyverse [47] (version 2.0.0) and rstatix [48] (version 0.7.2) packages.

### Statistical Analysis

Significant differences were determined using Prism (GraphPad Software) or the R rstatix package. Asterisks indicate statistical significance (*, *P* < 0.05; **, *P* < 0.01; ***, *P* < 0.001; ****, *P* < 0.0001).

## Results

### Clinical Details

**Fig. 1:**
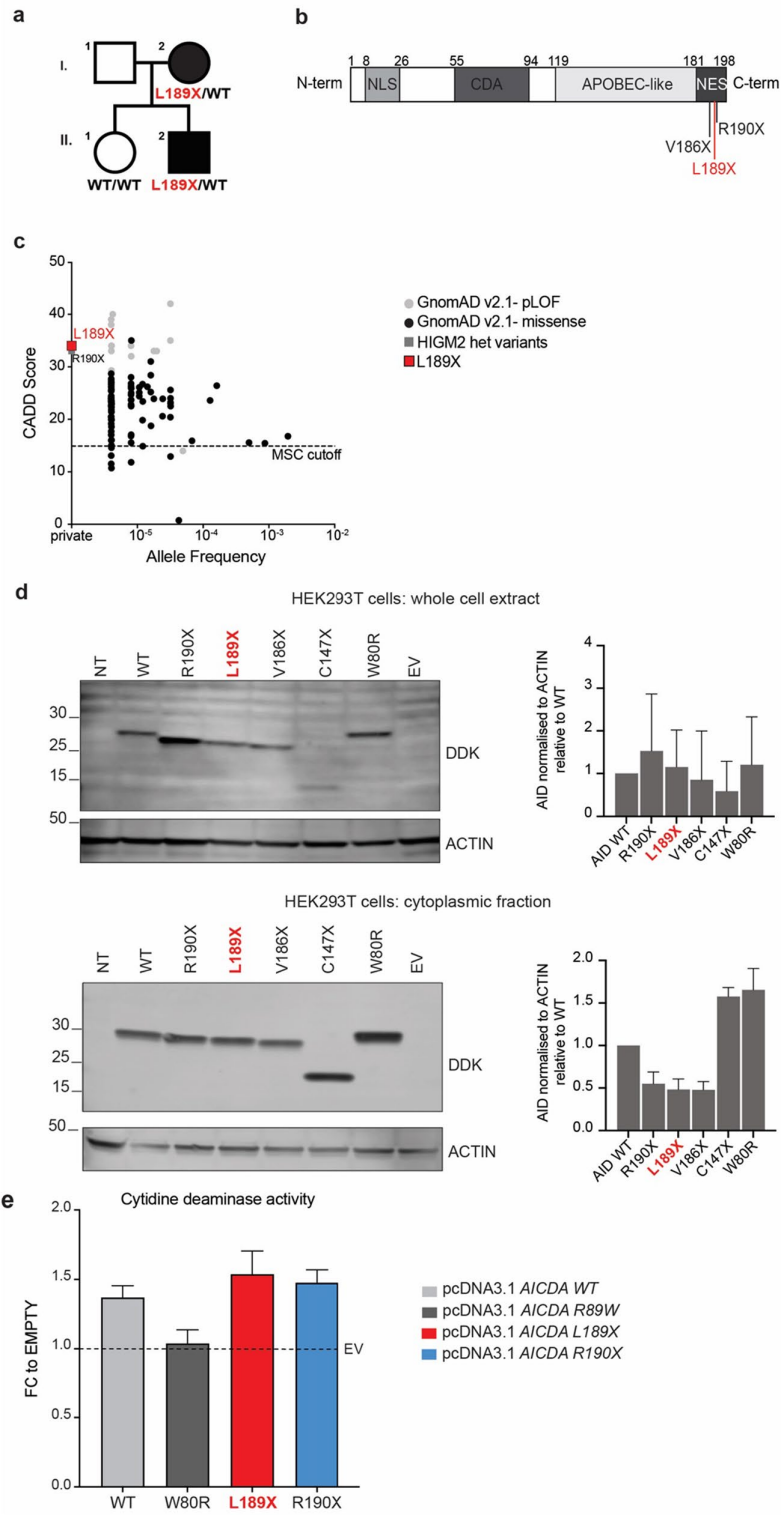
Identification of a novel heterozygous L189X/WT variant in *AICDA* in two related patients with hyper-IgM 2 (HIGM2) syndrome. **a** Pedigree showing familial segregation of the c.566_568delinsAA, p.L189X variant. Affected individuals are represented by closed black symbols (I.2, P1 and II.2, P2). **b** Schematic representation of AID protein showing its functional domains (NLS, nuclear localization signal; CDA, cytidine deaminase; NES, nuclear export signal; APOBEC, apolipoprotein B mRNA editing catalytic polypeptide-like), and location of heterozygous *AICDA* variants. **c** Minor allele frequency (MAF) and CADD score for predicted loss of function (pLOF, grey circles) and missense (black circles) *AICDA* variants reported in public databases, and variants found in previously-reported AD HIGM2 patients (grey square) and in P1 and P2 (red square). The mutation significance cutoff (MSC, 99% confidence interval) is represented by the dotted line. **d** HEK293T cells were untransfected (NT) or transfected with the DDK-tagged plasmids encoding WT or L189X AID (highlighted in red), or variants previously identified in patients with AD HIGM2 (V186X and R190X) [12, 30]; or C147X, located in the APOBEC-like domain [49]; W80R, previously shown to abolish AID enzymatic activity [50] or the empty vector (EV). Total cell extracts (upper panel) and cytoplasmic extracts (lower panel) were subjected to western blotting; anti-DDK antibody (Ab) was used to detect AID levels and anti-ACTIN Ab was used as a loading control. AID signal intensity for L189X (in red), R190X, V186X, C147X and W80R transfected cells relative to WT-transfected cells, in various cell compartments (total and cytoplasmic), were normalized against ACTIN, as shown by histogram bars graphs. The data shown are representative of three independent experiments. **e** HEK293T cells were untransfected (NT) or transfected with DDK-tagged plasmids encoding AID WT, AID L189X (highlighted in red), R190X, W80R, or the EV. Cell extracts were prepared and cytidine deaminase activity was determined. A fold-change (FC) of cytidine deaminase activity normalised to EV was calculated for each mutant protein. The data shown are mean ± SEM of four independent experiments

We studied a multiplex family comprising an affected mother (P1) and her son (P2). Prior to genetic testing P1 and P2 had been clinically diagnosed with common variable immunodeficiency (CVID; Table 1, Fig. 1a). Both individuals experienced early onset recurrent upper and lower respiratory tract bacterial infections, with P1 and P2 commencing intravenous Ig therapy (IVIg) at age 7 and 3 years respectively, with excellent clinical responses. P1 developed mild central bronchiectasis, gastrointestinal disturbance and has also been diagnosed with “seronegative” arthritis previously treated intermittently with methotrexate or leflunomide. P2 does not have bronchiectasis or arthritis. Neither individual has developed significant lymphadenopathy or splenomegaly, cytopenias, viral infections, atypical or opportunistic infections, malignancy or sclerosing cholangitis. They both have normal total lymphocyte counts, and serum IgM levels within the normal range, however serum IgG and IgA were undetectable in P1 wherease P2 had IgG2 subclass deficiency and low/normal IgA levels prior to commencing IVIg (Table 1). Interestingly, both P1 and P2 currently have undetectable levels of serum IgA (Table 1).

The index case (P1, I.2 on Fig. 1a) was enrolled in a study aimed at identifying disease-causing variants in a CVID cohort from Western Australia using a targeted NGS panel encompassing 120 IEI-causing genes [51]. This approach established that P1 carried a novel heterozygous variant in *AICDA* (c.566_568delinsAA) located in the NES domain at the C-terminal end of AID protein. Sanger sequence analysis showed that P1’s affected son (P2, II 2, Fig. 1a) also carried the same heterozygous variant whereas the daughter of P1 (II 1), who does not show any immunological disease, was WT for *AICDA* (Fig. 1a), thus confirming segregation with the disease phenotype. *AICDA* is a robust candidate disease-causing gene in this kindred as the clinical and laboratory features of HIGM and CVID are very similar. In fact, recurrent respiratory infections, hypogammaglobulinemia - except for IgM- and failure to Ig isotype switch are characteristic for HIGM patients and not unusual for CVID [52, 53].

In silico analysis predicted that c.566_568delinsAA introduces a premature stop codon at L189 (L189X), deleting the last ten amino acids of the NES domain and of AID protein (Fig. 1b). This variant is located between the V186X and R190X variants that have previously been found to be causal for AD AID deficiency [12, 29, 30]. The L189X variant is private to this family, being absent from public databases (gnomAD: https://gnomad.broadinstitute.org/, GMEVariome: http://igm.ucsd.edu/gme, TOPMedBravo: https://bravo.sph.umich.edu). Calculated combined annotation-dependent depletion (CADD) score for c.566_568delinsAA is 34, well above the mutation significance cutoff ((MSC) = 14.9) (Fig. 1c).

### AID L189X Variant does not Affect Protein Expression

To decipher the functional consequences of L189X variant, we adopted an overexpression system where HEK293T cells were transiently transfected with pcDNA3.1 vectors encoding WT AID or L189X AID detected in P1 and P2. As a comparison, we also tested several other mutant AID proteins: W80R, which was previously shown to be catalytically inactive [50]; C147X which is located in the APOBEC-like domain [49]; and V186X and R190X which are both located in the C-terminal NES domain and previously reported in patients with AD HIGM2 [29, 30]. All vectors included an N-terminal DDK tag to enable detection of the expressed AID protein. When total cell lysates were assessed, L189X, V186X and R190X AID variants identified in patients with HIGM2 where detectable at slightly lower molecular weights (MW) than WT (Fig. 1d). Consistent with the location of these variants, the pattern of migration of the mutant proteins was WT > R190X > L189X > V186X (Fig. 1d). AID C147X migrated at approx. 15-17 kDa, consistent with a predicted size of 17.66kDA, whereas W80R protein was detected at the same size as WT AID (Fig. 1d). Thus, the novel *AICDA* variant identified in P1 and P2 does not abolish protein expression, but does encode a truncated AID variant.

### AID L189X Truncated Protein is Depleted in Cytoplasmic but not Whole Cells Extract

In B cells, AID has been shown to predominantly localise (about 90%) in the cytoplasm as a result of an intricate balance between mechanisms of nuclear import, nuclear export, cytoplasmic retention and differential stability of the protein in each compartment [54, 55]. Interestingly, previous studies showed that AID R190X protein accumulated mostly in the nucleus [56]. Hence, it was suggested that C-terminal truncated AID is retained in the nucleus and exerts a dominant negative effect on WT AID [56]. Thus, we investigated intracellular localisation of the AID L189X truncated protein by testing AID expression in subcellular protein fractions (cytoplasm vs whole cell extracts) from transfected HEK293T cells (Fig. 1d). Immunoblot assessment revealed that the abundance of the mutant AID proteins R190X, L189X and V186X in total cell extracts was comparable to WT AID (Fig. 1d, upper panel). Interestingly, when compared to WT AID protein and normalised to housekeeping proteins, lower amounts of AID C-terminal mutant proteins R190X, L189X, V186X were detected in cytoplasmic extracts prepared from the same experimental condition (Fig. 1d, lower panel). These data thus establish that translation of the mutant plasmids is intact, and that AID L189X protein is not only depleted in the cytoplasm because it lacks the information for correct shuttle between cytoplasm and nucleus, but it also impacts intracellular localisation of the WT protein, at least in our current model.

### AID L189X Protein has Intact Catalytic Function In Vitro

We next tested whether the L189X truncating variant affected the enzymatic activity of AID by assessing its ability to catalyse deamination of dC to dU. For this purpose, a cytidine deaminase activity assay was performed (Fig. 1e). Briefly, whole cell lysates were prepared from HEK293T cells transfected with WT and various mutated *AICDA* plasmids; each lysate was incubated with a cytidine substrate that, in the presence of cytidine deaminase (from the cell lysate), is converted into uridine and NH_3_, as intermediates. The fold changed (FC) of the cytidine deaminase activity compared to cells transfected with an empty vector (EV) was calculated for each AID protein tested. This assay demonstrated that cells transfected with WT AID exhibited an increase of enzymatic activity over mock transfected cells (FC = 1.4), while the W80R variant induced no change, indicating this variant is catalytically inactive and disrupts enzymatic activity of AID, (FC = 1.04). Interestingly, catalytic activity of both the L189X and R190X AID variants was similar to WT AID, suggesting neither of these mutations affect AID cytidine deamination activity.

### Effect of the *AICDA* L189X Variant on T Cell Differentiation In Vivo and In Vitro

**Fig. 2:**
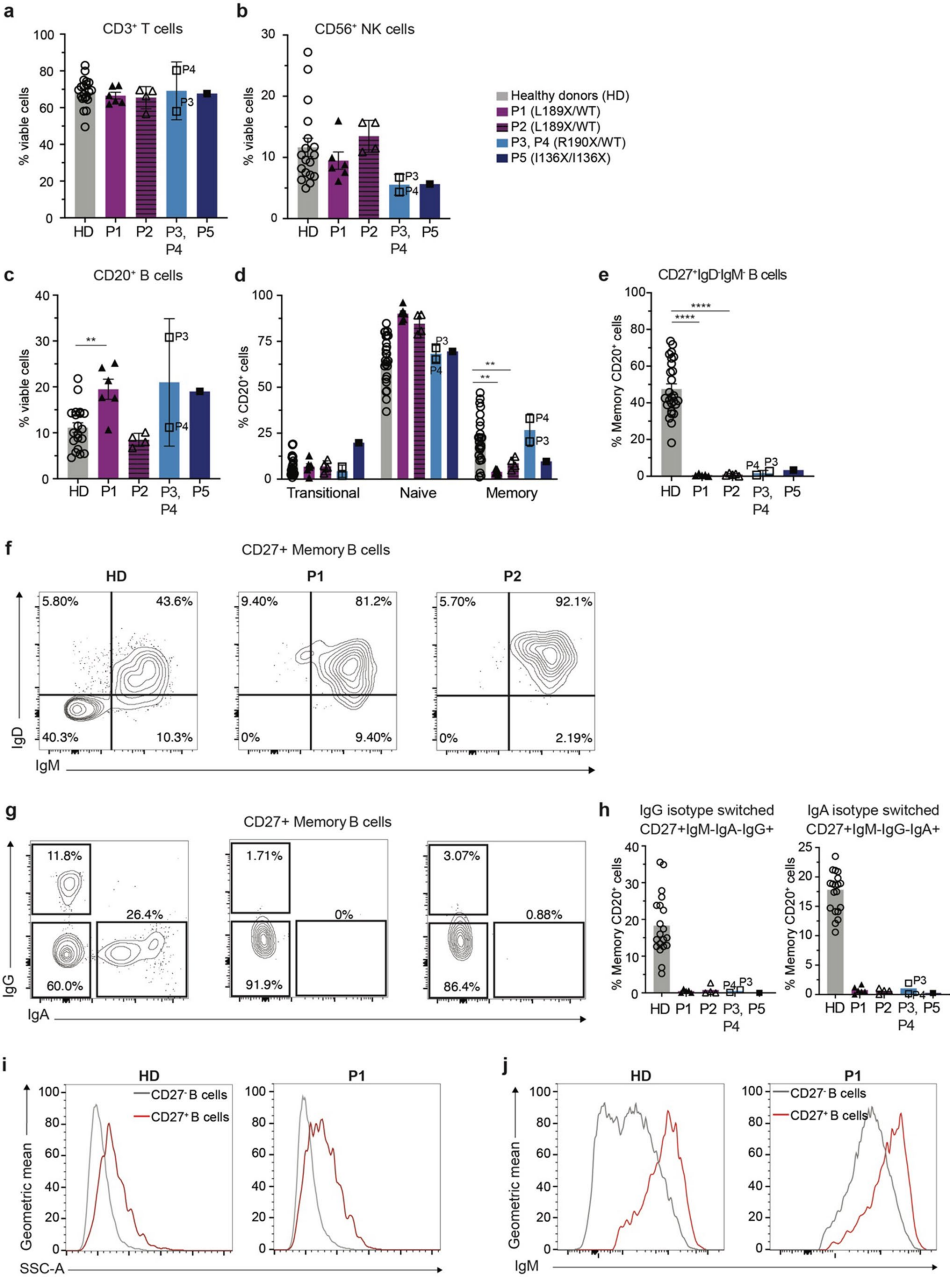
Dominant or recessive variants in *AICDA* disrupt memory B cell formation and Ig isotype switching. **a-e** PBMCs from healthy donors (HD, n = 16), P1 and P2 (heterozygous AID L189X variant), P3 (AID R190X/WT) [31], P4 (heterozygous AID R190X variant) and P5 (homozygous AID I136X variant) were stained to determine the proportions of (**a**) T cells (CD3^+^), **b** NK cells (CD56^+^) and (**c**) B cells (CD20^+^). Proportions of (**d**) transitional (CD27^−^CD10^+^), naïve (CD27^−^CD10^−^) and memory (CD27^+^CD10^−^) B cells and (**e**) Ig switched memory B cells (IgD^−^IgM^−^CD27^+^) were also determined. **f**, **g** Representative contour plots gated on HD, P1 or P2 showing frequencies of (**f**) IgD^+^, IgM^+^ or (**g**) IgG^+^, IgA^+^ CD27^+^ memory B cells. **h** Frequency of IgG^+^ or IgA^+^ switched memory B cells as determined by cell surface staining. **i, j** Characterisation of CD27^−^ B cells compared to CD27^+^ B cells identified in HD and AID deficient patients. Representative histogram plots gated on HD or P1 CD27^−^ (grey) and CD27^+^ (red) B cells showing the geometric mean of (**i**) SSC (90° light/side scatter), as a measure of cellular granularity, and (**j**) IgM surface expression. Each data point corresponds to individual healthy donors or AID-deficient patients; mean ± SEM are also shown. Results combined from at least three independent experiments. Statistical significance was determined by Mann–Whitney test, *P < 0.05, **P < 0.01, ***P < 0.001, ****P < 0.0001

Next, we characterised the impact of AID L189X variant on the phenotype of peripheral blood mononuclear cells (PBMCs). For this purpose, PBMCs from the index patient (P1) and her son (P2) carrying the AID L189X heterozygous variant, a previously-published patient heterozygous for the R190X AID variant (P3; R190X/WT) [31], an unrelated patient also heterozygous for the R190X *AICDA* variant (P4; R190X/WT), a patient with AR HIGM2 homozygous due to a homozygous *AICDA* variant (P5, I136X/I136X), and healthy donors (HD) were examined. PBMC samples collected at multiple time points were immunophenotyped for P1 and P2 (ages between 55–58 and 23–26 years old repectively), whereas only one PBMC sample was available for P3 (age 60 years), P4 (age 36 years old) and P5 (age 12 years old). P1 and P2 had normal proportions of total T cells, CD4^+^ and CD8^+^ T cells and NK cells (Fig. 2a, b and Fig S1). Tregs and subsets of CD4^+^ and CD8^+^ T cells (naïve, central memory, effector memory, T_EMRA_) and NK cells (CD56^hi/dim^) were also all largely within the normal range established from healthy donors (Fig S1). Both P1 and P2 had increased proportions of T follicular helpers (Tfh) cells, representing 17% and 11% of CD4^+^ T cells respectively compared to ~ 5% found in healthy donors (Fig S1). This is consistent with previously-reported AD AID-deficient patients [27].

To further our understanding of potential effects of AID L189X on CD4^+^ T cell function, we sort purified naïve (CD45RA^+^CCR7^+^) and memory (CD45RA^−^) CD4^+^ T cells from healthy donors, P1, P2 (L189X/WT) and P5 (I136X/I136X), and stimulated them with TAE (anti-CD2/CD3/CD28 mAb) beads alone or under different polarising conditions for 5 days and compared expression and secretion of Th1 (IFNγ, TNFα), Th2 (IL-4, IL-5, IL-13), Th9 (IL-9), Th17 (IL-17A, IL-17F, IL-22) and Tfh (IL-21) cytokines. Production of these cytokines by naïve and memory CD4^+^ T cells isolated from P1, P2 and P5 did not differ from their CD4^+^ T cell counterparts from healthy donors (Fig S2, Fig S3). Hence, consistent with intact proportions of memory- and effector-phenotype cells ex vivo, differentiation and function of naïve and memory CD4^+^ T cells are unaffected by the L189X *AICDA* variant identified in the patients (P1, P2) under study.

### Memory B Cell Generation and Ig Class Switching In Vivo Are Severely Compromised by the Heterozygous L189X *AICDA* Variant

AID protein is most highly expressed in GC B cells [57]. For this reason, we next performed in-depth analysis of B cells in patients with novel (P1, P2) and known heterozygous (R190X; P3, P4) or homozygous (I136X/I136X, P5) variants in *AICDA*. Proportion of CD3^−^CD20^+^ total B cells were elevated in P1 (mean ± SEM: 20.4 ± 2.5% of viable lymphocytes), P3 (30.8%) and P5 (19.1%), while proportions in P2 (8.9 ± 0.44%) and P4 (11.16%) were comparable to healthy donors (11.2 ± 0.86%) (Fig. 2c). We further investigated the B cell compartment by quantifying frequencies of transitional (CD27^−^CD10^+^), naïve (CD27^−^CD10^−^) and memory (CD27^+^CD10^−^) cells. Transitional B cells were similar to healthy donors, while naïve B cells were generally increased in all patients (Fig. 2d). P1 and P2 both had a paucity of memory B cells (mean ± SEM: 4% and 8.6%, respectively) compared to healthy donors (22 ± 2.7%). Fewer memory B cells were also found in P5 (I136X/ I136X; 9% of total B cells). In contrast, memory B cells represented respectively 20% of total CD20^+^ B cells in P3 (R190X/WT), in accord with data previously reported for this patient [31], and 30% of total CD20^+^ B cells in P4 (R190X/WT). Despite being able to detect some memory B cells in all AID-deficient patients, irrespective of *AICDA* variant, < 1% of memory B cells in P1 or P2 had undergone class switching, evidenced by an absence of CD27^+^IgD^−^IgM^−^ switched B cells (Fig. 2e, f). An inability of B cells from P1 and P2 to undergo Ig class switching was further confirmed by the inability to detect any IgG^+^ or IgA^+^ memory B cells in these patients (Fig. 2e, g, h), as well as the observation that > 80% of memory B cells exhibited an unswitched IgM^+^IgD^+^CD27^+^ phenotype (Fig. 2f). P3, P4 (R190X/WT) and P5 (I136X/I136X) also had extremely low frequencies of Ig class switched B cells compared to healthy donors (2.68%, 0.6% and 3.34% respectively, vs 47%; Fig. 2e).

To gain further insight into the nature of the few CD27^+^ memory-type B cells detected in P1 and P2, we first compared their size (using FSC parameter) and granularity (using SSC parameter). Memory B cells in P1 and P2 were 1.14 fold larger and 1.39 fold more granular than corresponding naïve B cells, very similarly to these morphological features of memory B cells from healthy donors (Fig. 2i, Fig. S2b) and consistent with previous reports [58–60]. Second, the residual CD27^+^ B cells detectable in P1 and P2 were found to express increased levels (geometric MFI) of IgM and and lower levels of IgD relative to their corresponding CD27^−^ naïve B cells, similar to naïve and memory B cells from healthy donors (Fig. 2j, Fig. S2c). Thus, these data confirm the CD27^+^ B cells detectable in AID deficiency are unswitched memory B cells. Hence, the novel *AICDA* L189X variant identified in P1 and P2 abolished the ability of B cells to undergo Ig class switching in vivo.

### Intact Proliferation but Impaired Ig Class Switching and Differentiation of *AICDA* L189X B Cells In Vitro

**Fig. 3:**
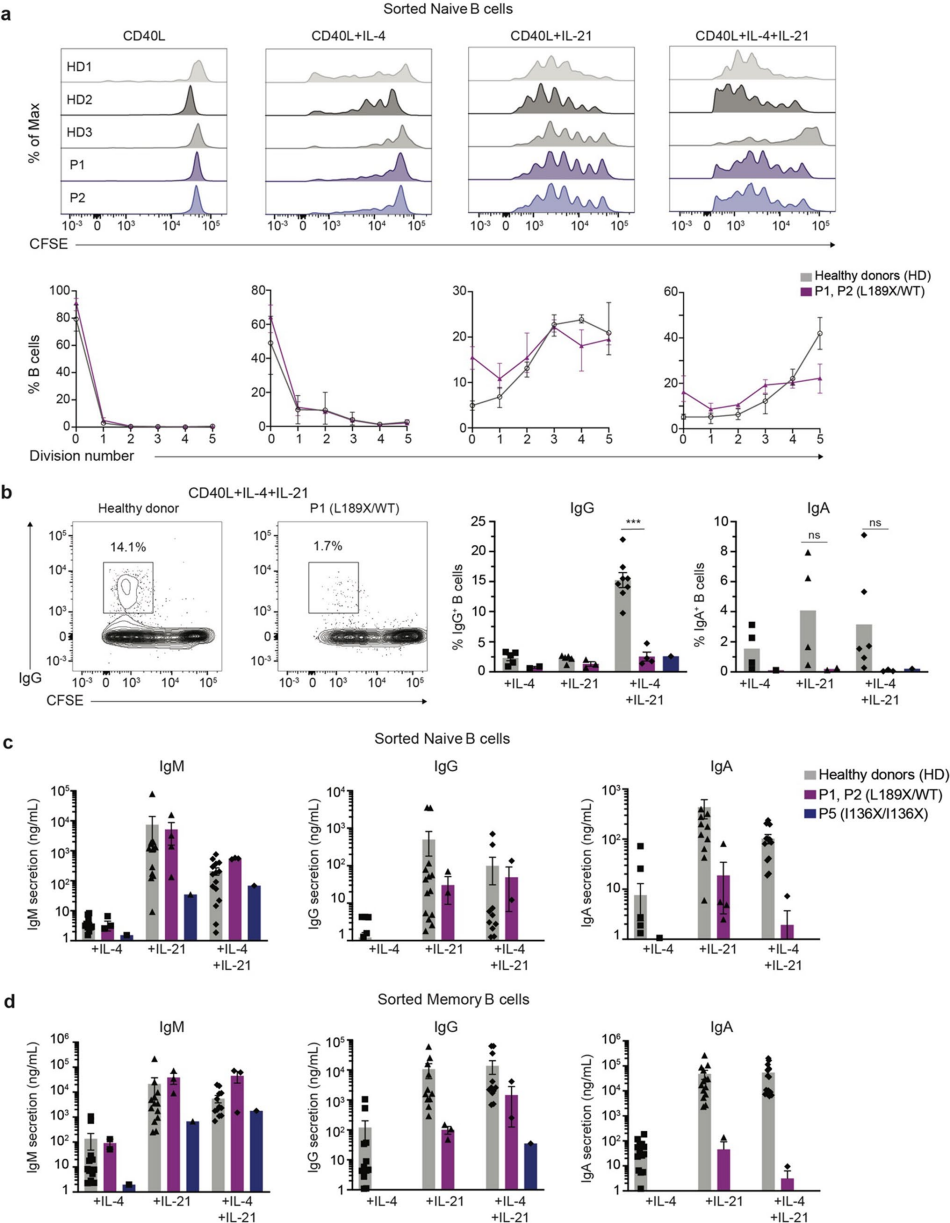
In vitro Ig switching and Ig secretion are impaired by the heterozygous AID L189X variant. (a-d). Sort-purified naïve B cells from healthy donors (HD, n = 3–13), P1 or P2 (heterozygous AID L189X variant) and P5 (homozygous AID I136X variant) were CFSE-labelled and cultured with CD40L alone or in combination with IL-4 (100 U/ml), IL-21 (50 ng/ml), or IL-4 and IL-21 for 5 days. After this time, cells were harvested and labelled with Zombie Aqua and mAb against IgG and IgA, followed by flow cytometric analysis. (**a**, upper panel) Histograms showing CFSE dilution of B cell from three healthy donors (HD1-HD3), P1, and P2 from one representative experiment. (**a**, lower panel) CFSE analysis for naïve B cells from healthy donors (n = 13) and P1 and P2 (n = 3), showing the frequency in each cell division interval. Values represent the means ± SEM of three independent experiments. (**b**, left panel) Representative contour plots from one HD and P1 showing the frequencies of IgG^+^ switched B cells vs proliferation (CFSE). (**b**, right panel) Percentage of IgG^+^ and IgA^+^ switched cells in cultures of naïve B cells from HD or patients with AID deficiency. Values depict data from individual HD and patients, and mean ± SEM. Statistical significance was determined by Mann–Whitney test, *P < 0.05, **P < 0.01, ***P < 0.001, ****P < 0.0001. **c-d** Sort-purified naïve (**c**) and memory (**d**) B cells from healthy donors (n = 10), P1 and P2 (n = 3) and P5 (n = 1) were cultured with CD40L and either IL-4 (100 U/ml), IL-21 (50 ng/ml), or IL-4 and IL-21. After 7 days, the amount of IgM, IgG and IgA secreted into the culture supernatant was measured by ELISA. Each point represents a different individual. The data shown are representative of three independent experiments, bars show median and interquartile ranges. Statistical significance was determined by Mann–Whitney tests, *P < 0.05, **P < 0.01

To confirm that the *AICDA* L189X variant intrinsically disrupts B-cell differentiation, we investigated in vitro responses of sort-purified naïve (CD10^−^ CD27^−^) and memory (CD10^−^ CD27^+^) CD19^+^ B cells from patients P1, P2 (L189X/WT) and P5 (I136X/I136X) and healthy donors following activation with a variety of stimuli (CD40L, CD40L + IL-4, CD40L + IL-21, CD40L + IL-4 + IL-21) that are well-characterised to induce proliferation, class switching, plasma cell generation and Ig secretion [61]. First, CFSE dilution was used to measure naïve B cell proliferation following a 5 day culture period. Whilst CD40L stimulation induced only modest proliferation in naïve B cells from healthy donors, the extent of cell division was greatly increased in the presence of IL-4, IL-21 or both cytokines (Fig. 3a). Importantly, although CD40L in combination with these cytokines potently induces expression of AID [62], proliferation of naïve B cells from P1 and P2 was comparable to that of naïve B cells from healthy donors irrespective of the in vitro stimulus (Fig. 3a). Second, we tested B cell class switching and plasma cell differentiation in vitro in these same cultures by assessing acquisition of expression of surface IgG or IgA (Fig. 3b), and measuring levels of secreted Ig (Fig. 3c). Consistent with our previous findings [35, 62], stimulation of naïve B cells from healthy donors with CD40L and IL-4 or IL-21 induces the generation of a small but detectable population of IgG^+^ or IgA^+^ cells; the combination of CD40L, IL-4 and IL-21 synergistically enhanced switching to IgG (Fig. 3b, left panel). Strikingly, induction of IgG^+^ class switched B cells was strongly reduced (IgG: 2.9% in P1 and P2, 2.6% in P5 vs 15.5% in HD in CD40L + IL-4/IL-21 cultures), while the generation of IgA^+^ switched B-cells was essentially abolished (IgA: 0.2% in P1 and P2 and P5 vs 4% in HD) by the *AICDA* L189X variant (Fig. 3b, right panel).

When Ig secretion was measured after 7 days of culture of naïve B cells with polyclonal stimuli, levels of IgM were comparable for P1, P2, P5 and healthy donors (Fig. 3c). However, secretion of IgG and IgA by P1 and P2 naive B cells was strongly diminished (30-50-fold), but not abolished, compared to naïve B cells from healthy donors (Fig. 3c). In contrast, IgG and IgA secretion by P5 naïve B cells was abrogated (Fig. 3c). Lastly, despite the paucity of memory B cells in P1 and P2, we compared Ig production in vitro by memory B cells from patients P1, P2, P5 and healthy donors. Similar to naïve B cells, IgM secretion by memory B cells from P1 and P2 was comparable to healthy donors and to P5, yet secretion of IgG and IgA was > 100-fold lower than that detected for healthy donor memory B cells (Fig. 3d). Overall, these data confirm ex vivo findings of a severe B-cell intrinsic Ig isotype switching defect in P1 and P2 due to the *AICDA* L189X variant. Importantly, even though Ig class switching is regulated by cell division, this defect does not reflect a general inability of the patients B cells to respond to specific stimuli in vitro, as revealed by not only intact cell division (Fig. 3a) but also secretion of IgM (Fig. 3c). Thus, these findings establish that the last ten residues of the C-terminal domain of AID are essential for isotype switching because heterozygous variants that disrupt this region of AID are sufficient to abolish CSR in vitro and in vivo.

### *AICDA* Variants Impair the Generation of Vaccine-Induced Ag-Specific B-Cell Responses

Production of specific antibodies against tetanus, diphtheria and pneumococcal Ags following vaccination was completely impaired in P2 (L189X/WT) (Table 1). This is reminiscent of patients with biallelic *AICDA* variants who lack anti-tetanus IgG antibodies [23]. However, P3 (R190X/WT) was previously reported to have protective levels of IgG antibodies against tetanus toxoid and *Streptococcus pneumonia* serotypes [31]. To further analyse the impact of AID deficiency on B cell responses following natural infection and/or immunization, we collected PBMCs from healthy donors or patients, P1, P2 (both L189X/WT), P4 (R190X/WT) or P5 (I136X/I136X) prior to and following vaccination against and/or infection with SARS-CoV-2 and assessed the generation SARS-CoV2-spike specific B cells. P1 had one confirmed mild infectious episode with SARS-CoV2 infection. P2 has been infected twice with SARS-CoV2, and required antiviral treatment (Paxlovid) but no hospital admission. P4 had also experienced two acute SARS-CoV2 infections and was treated with monoclonal anti SARS-CoV2 antibodies (Txagevimab/ Cilgavimab). In contrast, to the best of our knowledge, P5 remains uninfected (at least prior to the most recent blood collection).

**Table 1:** Clinical and immunologic features of the AID L189X/WT patients (P1 and P2)

	**P1**	**P2**
Year of birth	1965	1997
Sex	F	M
*AICDA* variant	L189X	L189X
Failure to thrive as newborn	No	No
Age commenced IVIg (Years)	7	4
Laboratory parameters		
IgG*	Unknown	Normal
IgM	Low/Normal	Low/Normal
IgA (current)	Undetectable	Undetectable
IgE (curent)	Undetectable	NT
Neutrophil count	Normal	Normal
Total Lymphocyte count	Low/Normal	Low
Total CD19 B cell proportions	Normal	Normal
Switched Memory B cell proportions (IgD-, CD27 +)	Undetectable	Undetectable
CD4 T cell proportions	Normal	Normal
CD8 T cell proportions	Normal	Normal
CD16/56 NK cell proportions	Normal	Normal
Anti-Tetanus Ab**	Absent	Absent
Anti- diphtheria Ab**	﻿Absent	Absent
Anti- Streptococcus pneumonia IgG**	﻿Absent	Not protective
Clinical features		
Recurrent Upper and Lower Respiratory Tract Infections	Y	Y
Bronchiectasis	Y	N
PJP Infection	N	N
Lymphadenopathy/Splenomegaly	N	N
GIT disturbance	Y	N
Arthritis (Seronegative)	Y	N
Liver disease or Sclerosing Cholangitis	N	N
Cryptosporidium infection	N	N
Recurrent or atypical viral infections	N	N
Fungal infections	N	N
Mycobacterial Infections	N	N
Autoimmunity	N	N
Cancer	N	N
Clinical response and good QOL on IVIg therapy	Y	Y

* = Prior to commencing IVIg, ** = After immunization, NT = Not tested, Y = yes, N = no; Both patients are documented to have no serological response to any vaccines including pneumococcal, tetanus and diptheria vaccines

The following patient blood samples were available for analysis:

- P1: 2 months, 15 months and 20 months following receipt of the 3rd SARS-CoV-2 vaccination. The latter two samples were also ~ 11 and 16 months post infection, respectively;
- P2: 2 months and 5 months following the 3rd SARS-CoV-2 vaccination, with the latter sample also being ~ 4 weeks post natural infection with SARS-CoV-2;
- P4: 2 weeks and 3 months following the 2.^nd^ SARS-CoV-2 vaccination (prior to natural infection with SARS-CoV-2)
- P5: 9 months following the 2nd SARS-CoV-2 vaccination.

**Fig. 4:**
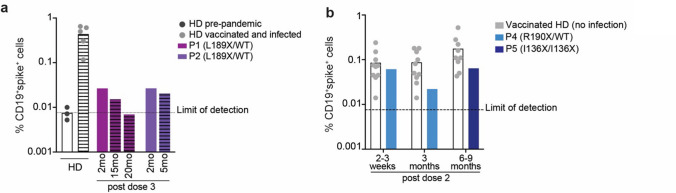
Impaired generation of spike SARS‐CoV‐2 B‐cells in AID deficient patients. PBMCs from healthy donors (HD) and AID deficient patients P1, P2 (AID L189X/WT), P4 (R190X/WT) and P5 (I136X/I136X) were collected at different time points following vaccination against and/or infection with SARS-CoV-2 and incubated with full-length SARS-CoV-2 Spike protein to detect spike-binding B cells (CD19^+^spike^+^). **a** Frequency of total Spike-binding B cells in pre-pandemic healthy donors (HD, dark grey circles), SARS-CoV-2 vaccinated and infected healthy donors (HD, light grey circles), P1 (L189X/WT, purple bars) and P2 (L189X/WT, violet bars) at the indicated time points indicated following infection/vaccination. Striped bars represent samples after natural infections. **b** Frequency of total Spike-binding B cells in vaccinated HD (grey circles), P4 (R190X/WT, light blue bars) and P5 (I136X/I136X, dark blue bars) detected at different time following vaccination (2–3 weeks, 3 months and 6–9 months after 2nd dose vaccine). Dotted line represent the limit of detection. Each point represents a different individual (HD)

Thus, we compared the specific B-cell response in P1 and P2 to those from a group of healthy donors who were both vaccinated against and infected with SARS-CoV2 (Fig. 4a), while responses in P4 and P5 were compared to indivduals who were vaccinated prior to natural SARS-CoV2 infection (Fig. 4b).

In PBMC samples collected from healthy donors prior to the COVID-19 pandemic, < 0.01% of B cells bound SARS-CoV2 spike protein (Fig. 4a). This defined the limit of detection of the assay (Fig. 4a, b). The mean percentage of Spike-binding/SARS-CoV2 specific B cells in healthy donors 3–6 months post vaccination/infection ranged from 0.1–0.5% (Fig. 4a). In contrast, the frequency of spike-binding B cells in P1 and P2 was 5–10-fold lower than in healthy donors, despite the patients being triple vaccinated (Fig. 4a). Interestingly, the frequencies of spike-binding B cells detected in P4 (R190X/WT) 2–3 weeks following receipt of two vaccine doses were comparable to healthy donors shortly (Fig. 4b). However, while these frequencies persisted or increased in healthy donors at later times post vaccination (3 mo), they promptly declined in P4 (Fig. 4b). At 9 months following the second vaccine dose, the frequency of spike-binding B cells in P5 (I136X/I136X) was lower but comparable to healthy donors (Fig. 4b). Unfortunately, no additional samples were available from P5 to assess persistence of SARS-CoV-2-specific B cells at later time points. Overall, these data demonstrate that *AICDA* variants severely impair the generation of Ag-specific B cells in vivo.

### Molecular Analysis of CSR and SHM in Patients B Cells

**Fig. 5:**
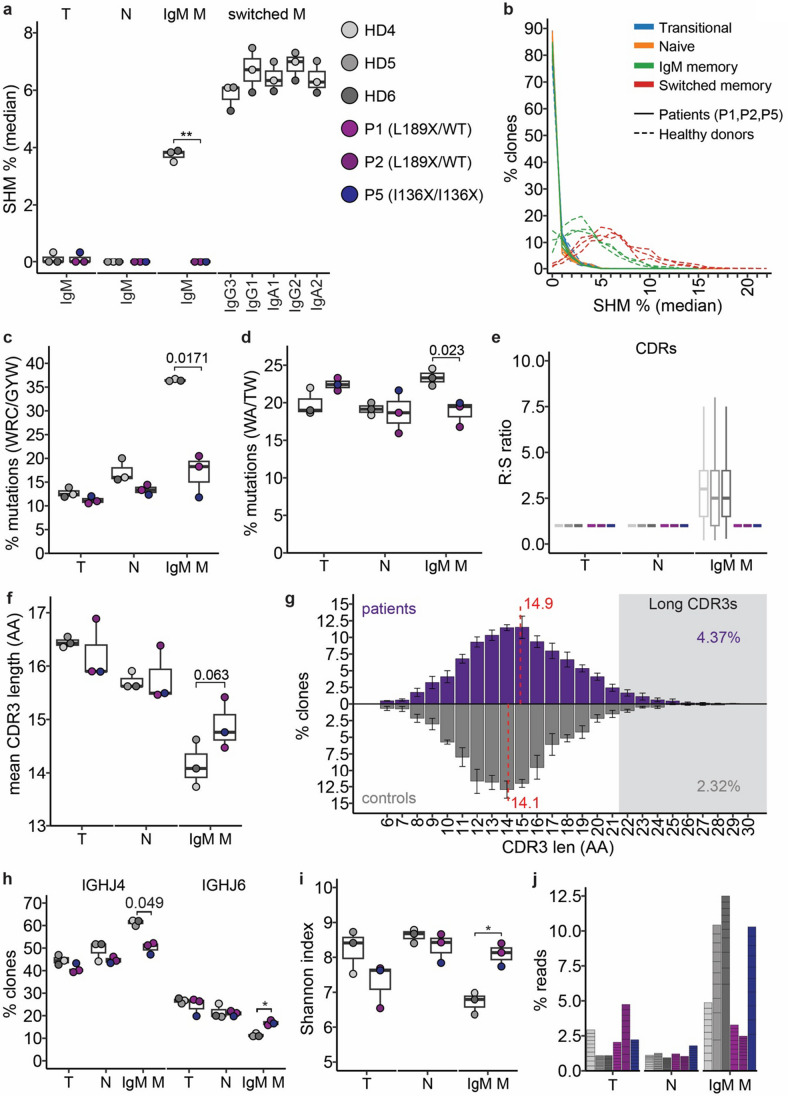
Heterozygous AID L189X variant dramatically disrupts somatic hypermutations (SHM). Deep sequencing of *IGHG*, *IGHA* and *IGHM* transcripts of transitional (CD27^−^CD10^+^), naïve (CD27^−^CD10^−^) and memory (CD27^+^CD10^−^) B cells from healthy donors (HD, n = 3), P1 (L189X/WT), P2 (L189X/WT) and P5 (I136X/I136X). **a** Median SHM% across all clones from transitional, naïve and memory (CD27^+^CD10^−^) B cells from HDs, P1, P2 and P5. Individual clones were summarise by median SHM from all reads. Filled circles show data for each individual and boxplots summarise the median, 25th/75th percentile and 1.5*IQR for each group (patient or HD) for each cell type/isotype. **b** Distribution of median SHM% for clones from each sample. Lines are coloured by cell type and group. **c** Frequency of mutations occurring at WRC/GYW motifs in transitional, naïve and memory IgM. Data from pooling all mutations from each individual using a representative sequence from each clone. Filled circles show data for each individual and boxplots summarise each group. **d** Frequency of mutations occurring at WA/TW motifs in transitional, naïve and memory IgM. Data from pooling all mutations from each individual using a representative sequence from each clone. Filled circles show data for each individual and boxplots summarise each group (**e**) Ratio of replacement (R, non-synonomous) to silient (S, synonomous) amino acid (AA) changes in transitional, naïve and memory IgM for each individual. Boxplots summarise R:S ratios in clone representatives. Outliers are not plotted. **f** Mean AA lengths of complementarity-determining region 3 (CDR3) for clones from each individual for transitional, naïve and memory B cells. Filled circles show data for each individual and boxplots summarise group. **g** Distribution of CDR3 lengths for patients (upper) and healthy donors (lower) within the IgM + memory compartment. Each bar indicates the mean % clones for the AA length and error bars show standard deviation. Overall mean CDR3 length for each group indicated by dashed red line. The proportion of clones with long CDR3s (> 22AAs) is indicated. **h** Frequency of clones with *IGH* rearrangements using *IGHJ4* or *IGHJ6* for transitional, naïve and memory IgM B cells. Filled circles show data for each individual and boxplots summarise each group. **i** Shannon Index (H) as a measure of clone diversity. Filled circles show data for each individual and boxplots summarise each group. **j** Clone sizes for ten largest clones from each sample. Each clone is represented as a stacked section that indicates the clone size as a percentage of total reads from the sample. Bars are coloured for the individual

To further characterise the consequences of AID variants on B-cell differentiation, we investigated CSR and SHM at the molecular level in transitional, naive, and memory B cells from P1, P2 (L189X/WT), P5 (I136X/I136X) and healthy donors by performing deep sequencing of *IGHG*, *IGHA* and *IGHM* transcripts. Amplification of *IGHG* and *IGHA* sequences was only achieved using B cells from healthy donors (not shown), thus confirming the flow cytometric analysis of patient B cells and serum Ig levels, which showed CSR was completely impaired in patients with either the L189X/WT heterozygous or I136X/I136X homozygous *AICDA* variants (Table 1, Figs. 2e-h and 3b). For this reason, we focused analysis of SHM on *IGHM* transcripts only. SHM is an AID-dependent process necessary for Ag-specific affinity maturation of Ig in the GC [63], thus the frequency of SHM increases as naïve B cells differentiate into memory B cells. As expected SHM was absent in the *IGH* repertoires of IgM^+^ transitional and naïve B cells from both patients and healthy donors (median rate of SHM in both B-cell subsets: 0%, Fig. 5a). As expected, the level of SHM in *IGHM* transcripts amplified from IgM^+^ memory B cells from healthy donors was significantly increased compared to transitional and naïve B cells (median SHM 3.83%, range 3.50—3.89%, Fig. 5a). Strikingly, the rate of SHM in *IGHM* transcripts amplified from memory B cells isolated from *AICDA*-deficient patients remained comparable to that of unmutated transitional and naïve cells (median SHM: 0% for P1, P2 and P4, Fig. 5a).

A fraction of IgM memory clones (mean 6.67%) from *AICDA*-deficient patients exhibited more than 2% of nucleotide mismatches compared to germline sequences; in contrast, an average of 76.47% of IgM^+^ memory B cells from healthy donors exhibited > 2% of SHM. The underlying distribution of mismatches for the patients was highly skewed to < 2% among the IgM^+^ memory B cells (Fig. 5b). This was essentially identical to the distribution of mismatches in transitional and naïve B cells from healthy donors and the patients, but was in stark contrast to the range of SHM for IgM^+^ memory B cells from healthy donors (Fig. 5b). The distribution of mutations between the complementarity determining regions (CDRs) and framework regions (FRs) also differed for the IgM^+^ memory B cells from patients and healthy donors. On average, healthy donors accumulated 35.0% of mutations in CDRs and 65.0% in FRs, however among the patients only 14.5% of mutations were concentrated in CDRs with 85.5% in FRs. Again, this mirrors the distribution of mutations in naïve (86.7% and 85.0% in FRs for patients and healthy donors, respectively) and transitional (86.6% and 89.2% in FRs) B cells.

A hallmark of AID-directed SHM is the accumulation of mutations at GYW/WRC motifs [22]. Patient and healthy donor B cell compartments retained a similar frequency of ‘mutations’ at AID hotspots for transitional and naïve B cells with on average 11.0% for patients and 12.4% for healthy donors for transitional (*p* = 0.11, t-test, Bonferroni corrected) and 13.3% compared to 16.0% (*p* = 0.10) within the naïve compartment (Fig. 4c). Within the IgM^+^ memory compartment, healthy donors B cells accumulated on average 36.3% of mutations at WRC/GYW hotspots compared to 18.3% for patients (*p* = 0.017). Among the patients, P5 had just 11.8% of mismatches at AID hotspots, consistent with the transitional and naïve compartments, whereas P1 and P2 were slightly elevated at 18.3% and 20.5%, respectively (Fig. 5c). Repair of AID-induced lesions by non-canonical mismatch repair (ncMMR) involving error prone polymerase eta can result in mutations at WA/TW motifs proximal to the deamination position [64]. The signature of ncMMR is also lacking in IgM^+^ memory B cells from the AID deficient patients compared to healthy donors (Fig. 5d) whereby the patients maintain a similar proportion of mismatches at WA/TW to naïve B cells (mean 18.7% for both naïve and IgM^+^ memory) but healthy donors display a significant increase with on average of 23.4% mutations accumulating at these motifs (*p* = 0.023, t-test, Bonferroni corrected). SHM also alters the ratio of transition and transversion nucleotide changes. Patients maintained a similar frequency of transition mutations across all three compartments; on average 65.8%, 67.1% and 67.5% for transitional, naïve and IgM^+^ memory, respectively. The healthy donors however demonstrated a significant decrease in transition mutations compared to the patients, reduced to 55.4% (*p* = 0.032, t-test, Bonferroni corrected).

Affinity maturation within the GC reaction exerts a selection for mutations that alter IGH specificity [65]. Under positive selection for Ag binding this skews the distribution of mutations such that the ratio of replacement (R, non-synonymous) to silent (S, synonymous) favours replacement mutations particularly in the CDRs. While this effect was clearly observed in IgM^+^ memory B cells from the healthy donors, it was completely absent from all three AID-deficient patients (Fig. 5e). Healthy donors exhibited a mean CDR R:S ratio of > 3 (HD4: 3.2, HD5: 3.0, HD6: 3.2), while this was on average 1.2 for IgM^+^ memory B cells for all three AID-deficient patients (*p* = 0.0038, t-test, Bonferroni corrected) which is similar to transitional and naïve compartments. For the FRs, the mean R:S ratio within transitional and naïve B cells was < 1.0 for both patients (mean R:S 0.80 and 0.91, respectively) and healthy donors (mean R:S 0.79 and 0.83, respectively). In contrast, for IgM^+^ memory B cells in healthy donors, the mean FR R:S ratio increased to 1.98, but remained signicantly lower for the patients (0.87; *p* = 0.0018, t-test, Bonferroni corrected).

Combined, these findings convincingly demonstrate that functionally the *AICDA* heterozygous (P1 and P2) and homozygous patients (P5, I136X/I136X) (Fig. 5a-e) display highly similar consequences to SHM within the IgM^+^ memory B cells, with approx 94% of clones remaining unmutated, and a lack of the signatures of AID-directed SHM and affinity maturation, thereby strongly resembling transitional and naïve B cells.

### Characterisation of Patients *IGH* Repertoire

Analysis of the non-SHM features of the *IGH* repertoire of AID-deficient patients revealed that IgM^+^ memory B cells have shorter CDR3 length (mean 14.88aa) compared to transitional (16.23aa) and naïve (15.78aa) B cells [66] (Fig. 5f). This was also observed for B cell subsets isolated from healthy donors, with CDR3 length decreasing from an average of 16.44 aa to 15.72 aa and then to 14.14 aa in transitional, naïve and memory B cells, respectively (Fig. 5f). The CDR3 length of IgM^+^ memory B cells from AID-deficient patients however showed less of a decrease between transitional to IgM memory, reducing by 1.35 aa compared to 2.30 aa for healthy donors, with patients on average having mean CDR3 length among IgM^+^ memory of 14.9 aa compared to 14.1 aa for healthy donors (Fig. 5f, g). Patients’ memory B cells also displayed a higher proportion of long CDR3s (22 aas or more) compared to healthy donor memory B cells (4.37% vs 2.32%, pooling clones for each group), a feature that favours antibody self-reactivity (Fig. 5g). Longer CDR3s may correlate with increased *IGHJ6* usage as *IGHJ6* has the potential to contribute more residues to CDR3s. As B cells move from transitional to IgM^+^ memory, healthy donors increased *IGHJ4* usage and decreased *IGHJ6* usage (Fig. 5h). However, *AICDA*-deficient patients’ IgM^+^ memory B cells retain a higher usage of *IGHJ6* accompanied by a lower increase in *IGHJ4* (Fig. 5h). *IGHJ4* mean usage for patients in IgM^+^ memory was 50.2% which was significantly lower than healthy donors (61.30%, *p* = 0.0493, t-test with Bonferroni correction), whereas *IGHJ6* usage remained at an average of 16.8% compared to 11.3% for healthy donors (*p* = 0.0202, t-test with Bonferroni correction).

When examining clonal diversity of IgM^+^ memory B cells, *AICDA* heterozygous (P1 and P2) and homozygous (P5, I136X/I136X) patients clustered together with a Shannon Index (H) similar to the transitional and naïve B-cell compartments which lack clonal expansions (Fig. 5i). In contrast, the Shannon index for IgM^+^ memory B cells from healthy donors was significantly lower than that of transitional and naïve B cells (*p* = 0.020, t-test, Bonferroni corrected) (Fig. 5i). Among healthy donors, the diversity of IgM^+^ memory B cells was reduced as a result of increases in clonally expanded B cells, whereas the patients—particularly P1 and P2—retained an even distribution of clones size across transitional, naïve and IgM^+^ memory B cell subsets (Fig. 5j). Overall, these data suggest that the repertoire of IgM^+^ memory B cells from patients with either AD- or AR-AID deficiency retain features of the less mature transitional and naive B cell compartments compared to IgM^+^ memory B cells from healthy donors.

## Discussion

Herein we report the impact on B lymphocyte development, differentiation and function of a novel pathogenic heterozygous *AICDA* variant identified in two related individuals. The patients’ clinical phenotype is characterised by recurrent infections responsive to intravenous Ig prophylaxis, normal serum levels of IgM but low to subnormal levels of IgG and undetectable IgA, and impaired Ag-specific humoral immune responses. Furthermore, patients’ B cells were unable to undergo Ig CSR and produce IgG and IgA in vitro in response to stimulation with CD40L and different cytokines. These findings are reminiscent of previously-published HIGM2 patients with predominantly AR, but also several AD, variants in *AICDA* [29–31].

Both P1 and P2 carry a novel heterozygous *AICDA* variant (c.566_568delinsAA) which introduces a premature stop codon at residue 189 (L189X), truncating the last 10 residues at the C-terminus of AID protein and loss of the NES. Our data showed that AID L189X protein is expressed at the expected lower molecular weight compared to WT AID, however it retains deaminase activity similar to WT. Thus, in contrast to biallelic *AICDA* variants that directly impact AID expression and/or its enzymatic activity, AID L189X preserves both. Therefore, AID L189X does not cause disease due to haploinsufficiency. Studies on AID WT protein subcellular localisation showed that a multi-layered dynamic equilibrium regulates its intracellular trafficking, and only 10% of endogenous AID partitions to the nucleus [55, 67]. While the significance of such complex regulation is not fully understood, it likely limits access of AID to the genome to ensure an optimal balance between Ig diversification and deleterious off-target activity [54]. Interestingly, only 2 other *AICDA* variants have been found to be pathogenic in a heterozygous state [12, 16, 29]. R190X AID was previously shown to aberrantly accumulate in the nucleus [56]. Similarly, we showed that AID L189X was depleted from the cytoplasm, suggesting that C-terminal heterozygous *AICDA* variants encode a protein that is retained in the nucleus, potentially interfering with AID WT by a dominant negative mechanism. Further studies on intracellular trafficking of different AD-AID mutant proteins might help in elucidating mechanisms of disease pathogenesis in the setting of AD-HIGM2 compared to impaired expression and/or function of AID as the cause of disease in AR-AID deficiency.

AD-AID patients carrying heterozygous *AICDA* variants affecting the last 9–13 residues of the C-terminal NES domain have been reported to uniformly present with impaired Ig CSR; in contrast the effect on SHM of Ig H chain genes is variable [12, 16, 30]. Our data showed that the AID L189X variant indeed abrogated CSR, consistent with the phenotype (lack of Ig class switched B cells ex vivo) and compromised function (impaired class switching in vitro) of B cells from individuals with previously-reported heterozygous *AICDA* variants [12, 16, 30]. However, we also found that AID L189X drastically affected, both quantitively and qualitatively, SHM. In fact, the frequency of SHM in IgM expressed by patients’ memory B cells was similar to transitional and naïve B cells, which are largely unmutated, and also to that of B cells from an AR-AID deficient patient. Thus, truncation of the last 10 residues of the NES of one copy of AID due to the L189X variant is sufficient to prevent AID from correctly mediating not only CSR but also SHM. Furthermore, the BCR repertoire of AD-AID L189X individuals was more diverse and had increased CDR3 length compared to healthy donors, features that generally characterise antibody polyreactive and autoimmunity [68]. Interestingly, neither P1, P2 nor any of the other AD-AID patients previously reported developed clinical autoimmune features, whereas this occurs in at least 25% of the AR-AID deficient patients due to defects in peripheral and central B-cell tolerance [69]. Our findings suggest that B-cell tolerance is not impacted in the AID L189X heterozygous patients, consistent with an absence of detectable autoreactive IgM in serum of other AD-AID deficient patients [27].

In healthy donors, enrichment of SHM in CDRs and an increased R/S ratio indicates effective affinity maturation of humoral immune responses [27]. Patients with AD-AID and AR-AID showed distinct features compared to healthy donors in terms of both localisation and pattern of the minimal level of SHM detected in patients’ B cells. Moreover, somatic mutations detected in AD-AID IgM^+^ memory B cells showed reduced targeting of hotspot motifs WRCY/RGYW and WA/TW, in line with a deleterious impact of L189X on AID-dependent SHM. The patients also showed a skewed mutational pattern with accumulation of R mutations in the CDR and FRs and higher frequency of transition mutations. In our hands, the targeting and pattern of residual SHM in AID L189X patients were very similar to those from AR-AID patient tested (P5, I136X/I136X), suggesting that both monoallelic truncation of the NES and biallelic variants in exons 1, 2, 3, or 4 of *AICDA* lead to similar qualitative defects of SHM.

At face value, our findings of drastically reduced, if not completely abolished, SHM in IgM^+^ memory B cells from patients with AID L189X contrast with initial studies that found SHM was intact in AD-AID patients [12, 16, 33]. However, a more recent study reported a 2.5–3 reduction in the frequency of SHM in *IGHM* in IgM^+^ memory B cells isolated from 4 patients with AD *AICDA* variants compared to corresponding cells from healthy donors [27]. Thus, it is possible that heterozygous variants affecting the C-terminal domain of AID do impact SHM, with the varying impact depending on the specific *AICDA* variant. This is supported by previous data showing that SHM in memory B cells from a patient with a heterozygous *AICDA* V186X variant was 5–6-fold lower than in healthy donors, while SHM in patients heterozygous for the R190X variant was reduced 2-fold [25]. This, together with our data, suggests that L189X, V186X, and R190X uniformly abolish CSR but have a variable impact on mutational frequency, with V186X and L189X seemingly more compromised than R190X. This is also consistent with our finding of detectable SARS-CoV2-specific B cells in P4 (R190X/WT) following vaccination, but very few Ag-specific B cells in P1 and P2. These important observations indicates that more in-depth analysis of SHM in AD AID deficiency – using similar approaches to what we have adopted here—is warranted to determine the full impact of these heterozygous C-terminal variants on affinity maturation of humoral immune responses in humans, and to determine whether differences in SHM due to distinct variants in *AICDA* alter clinical features of AD AID deficiency.

## Conclusion

In summary, we discovered that AID L189X protein, despite preserving expression and catalytic activity, has significant deleterious effects on Ig CSR and SHM, as well as memory B-cell generation. Moreover, intracellular localisation of AID L189X was compromised, suggesting the intrinsic enzymatic activity of AID might be modulated based on its location and association with CSR and/or SHM-specific cofactors. Therefore, the localisation and precise AID targeting potentially mediated by co-factors are likely to influence the phenotype of AID deficient patients. Further studies aimed to better understanding the intracellular trafficking of C-terminal truncated AID and the specific cofactors involved are required to determine the mechanisms of disease causation in patients with HIGM2 due to heterozygous *AICDA* variants.

### Supplementary Information

Below is the link to the electronic supplementary material.

Supplementary file1 (DOCX 1013 KB)

## Data Availability

Available upon request to the corresponding authors.
