# Supplementary material for: A Novel Heterozygous Variant in AICDA Impairs Ig Class Switching and Somatic Hypermutation in Human B Cells and is Associated with Autosomal Dominant HIGM2 Syndrome
Source: J Clin Immunol. 2024 Feb 16;44(3):66. doi: 10.1007/s10875-024-01665-1 (PMC10873450; doi:10.1007/s10875-024-01665-1)

**SUPPLEMENTARY INFORMATION**

**A novel heterozygous variant in *AICDA* impairs Ig class switching and somatic hypermutation in human B cells and is associated with autosomal dominant HIGM2 syndrome**

Erika Della Mina^1,2^, Katherine JL Jackson^1^, Alexander JI Crawford^1^, Megan L Faulks^1^, Karrnan Pathmanandavel^1,2^, Nicolino Acquarola^3^, Michael O’Sullivan^3,4^, Tessa Kerre^5^, Leslie Naesens^6^, Karlien Claes^7,8^, Christopher C Goodnow^1,2^, Filomeen Haerynck^7,8^, Sven Kracker^9,10^, Isabelle Meyts^11,12^, Lloyd J D’Orsogna^3,13,#^, Cindy S Ma^1,2,#^, Stuart G Tangye^1,2@^

**Index:**

**Figures S1, S2, S3 and legends**

**Supplemental figures legends**

**Figure S1**

PBMCs from healthy donors (HD, n=16), P1 and P2 (heterozygous AID L189X variant), P3, P4 (AID R190X/WT) and P5 (AID I136X/I136X) were stained to determine the proportion of (**A**) CD4^+^ and CD8^+^ subpopulations, (**b**) Tregs (CD25^high^CD127^low^) and (**c**) NK cells subsets (CD56^high^/CD56^low^) (**d-e**) Proportion of naive (CCR7^+^CD45RA^+^), central memory (CCR7^+^CD45RA^−^), effector memory (CCR7^−^CD45RA^−^), effector revertant memory (EMRA; CCR7^−^CD45RA^+^) CD4^+^ (**d**) or CD8^+^ (**e**) T cells were also determined. (**f**) Frequency of Tfh (CD25^low/−^CXCR5^+^CD45RA^−^) was also measured in non-Tregs cells. Each data point corresponds to individual healthy donors or AID-deficient patients; mean ± SEM are also shown. Results combined from at least three independent experiments. (**g**) Size and granularity of CD20^+^CD27^-^ (grey) and CD20^+^CD27^+^ (red) cells were determined by measuring (**top**) forward scatter (FSC) and (**bottom**) side scatter (SSC), respectively, by flow cytometry. The ratio of size and granularity of CD27^+^ to CD27^-^ B cells were determined (table below histograms). (**h**) Representative histograms plots gated on HD or P1 CD27^-^ (reported in grey) and CD27^+^ (reported in red) B cells showing surface expression of (top) IgM or (bottom) (IgD).

**Figure S2**

Sort-purified naive CD4^+^ T cells from healthy controls (n = 6) and P1 and P2 (heterozygous AID L189X, n=3) and P5 (homozygous AID I136X variant, n=1) were cultured for 5 days with TAE beads. (**a-e**) Intracellular expression of (**a**) Th1, (**b**) Th2, (**c**) Th9, (**d**) Th17 or (**e**) Tfh cytokines were determined by intracellular staining. Each data point corresponds to individual healthy donors or AID-deficient patients; mean ± SEM are also shown.

**Figure S3**

Sort-purified memory CD4^+^ T cells from healthy controls (n = 6) and P1 and P2 (heterozygous AID L189X variant, n=2), were cultured for 5 days with TAE beads. (**a-e**) Intracellular expression of (**a**) Th1, (**b**) Th2, (**c**) Th9, (**d**) Th17 or (**e**) Tfh cytokines were determined by intracellular staining. Each data point corresponds to individual healthy donors or AID-deficient patients; mean ± SEM are also shown.


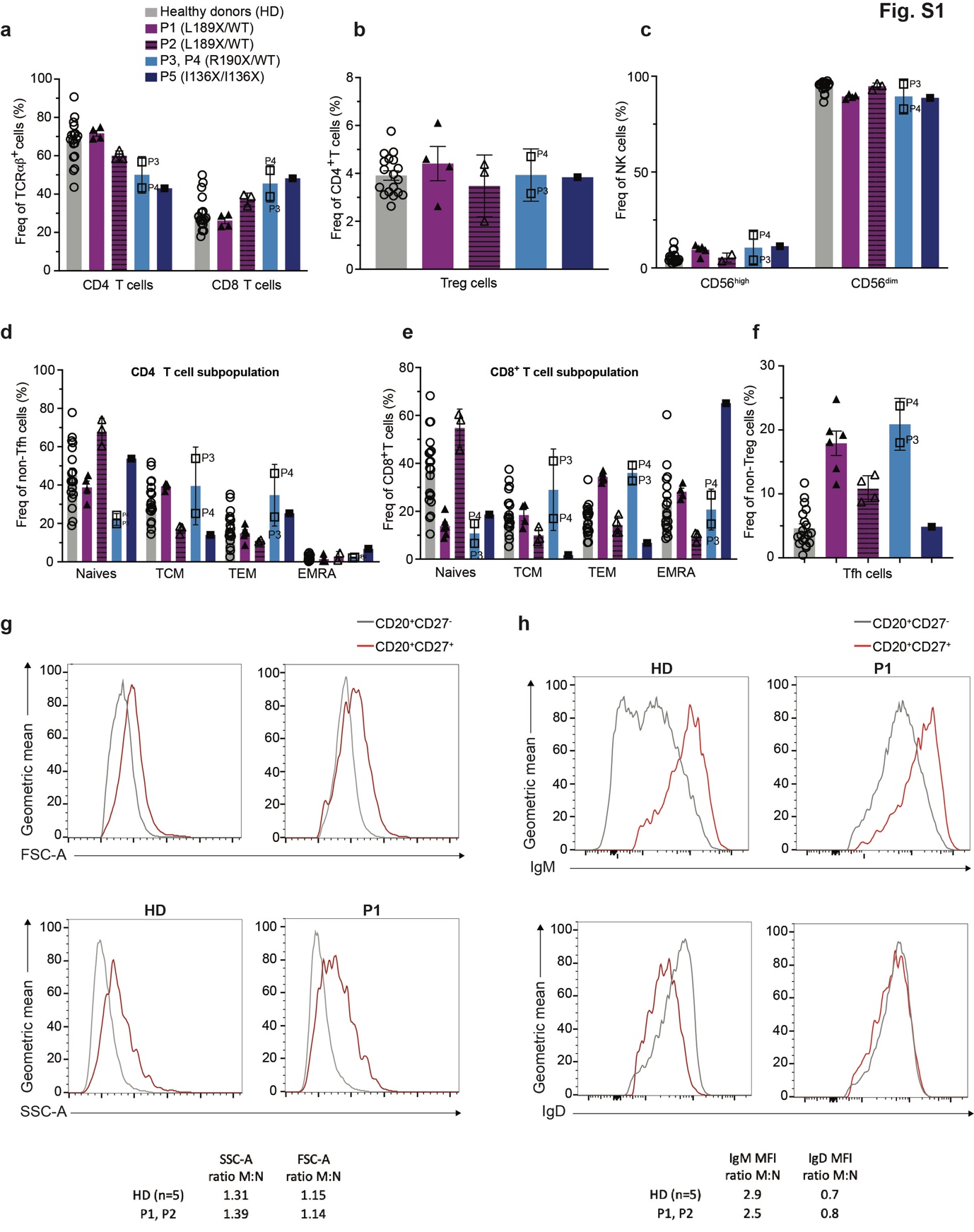


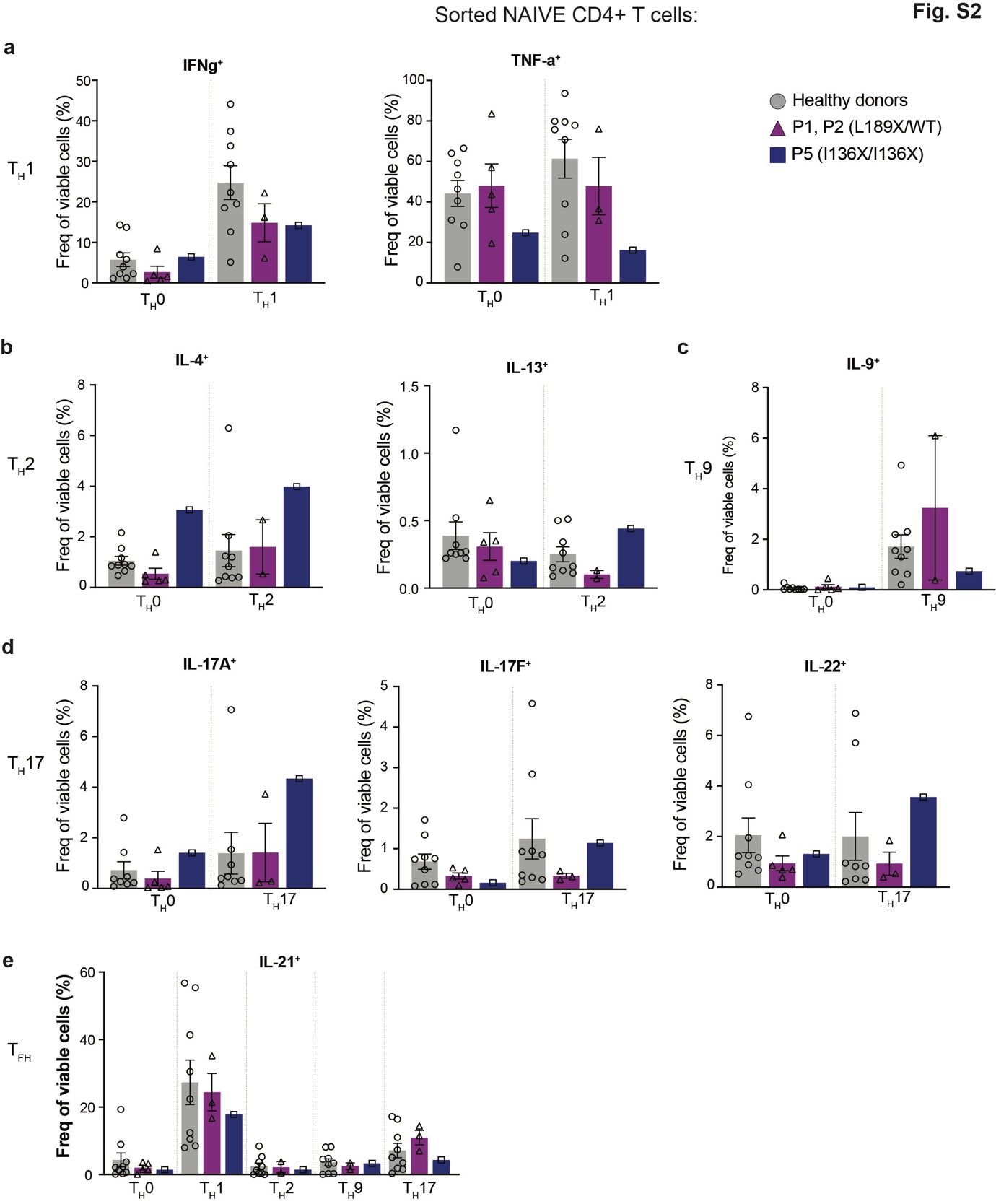


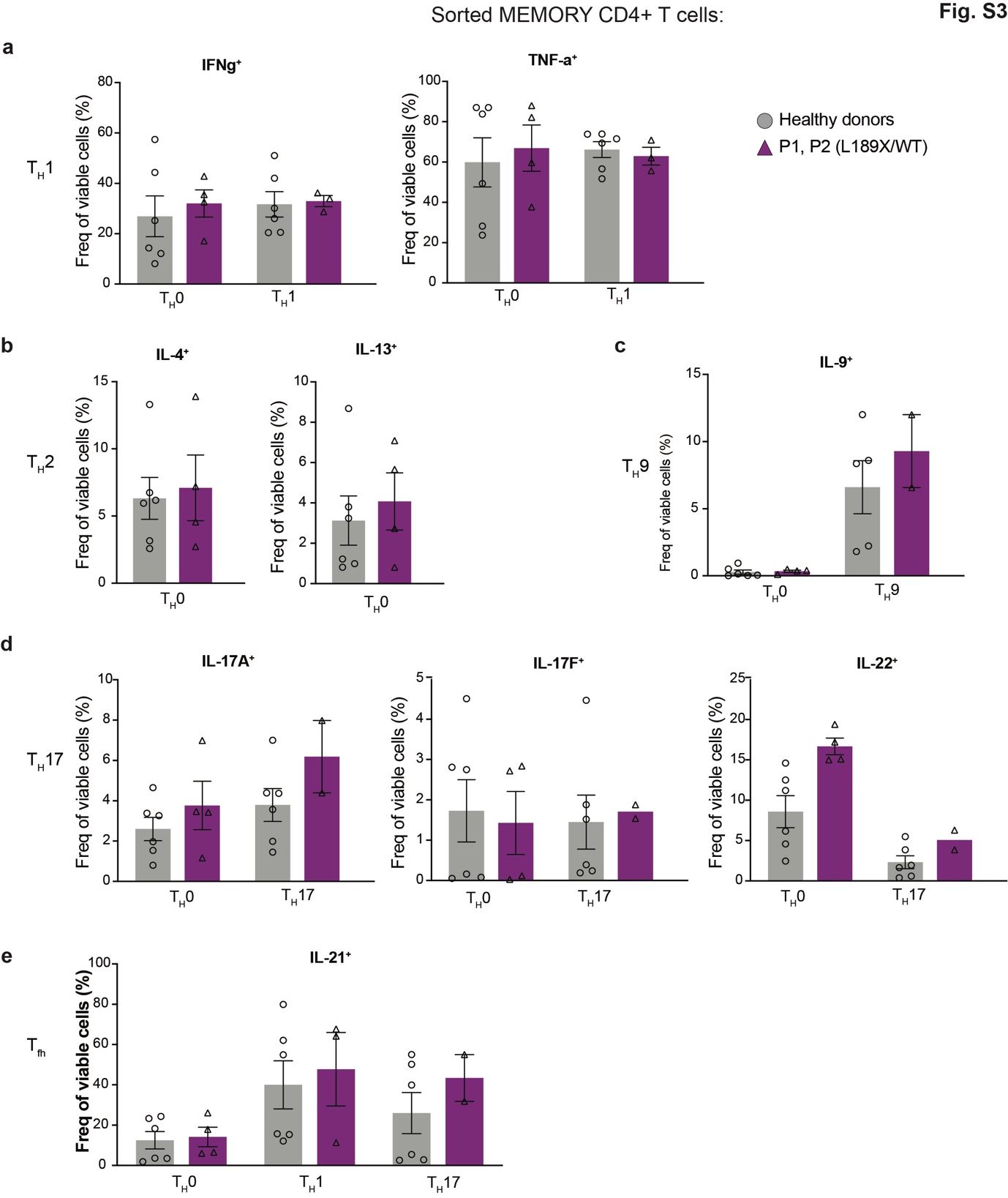

Supplement: Supplementary file 1 — Supplementary file1 (DOCX 1013 KB) [file 10875_2024_1665_MOESM1_ESM.docx]
